# COVID-19 vaccine uptake at six months post vaccine availability in Central Texas: an observational study disentangling the moveable middle

**DOI:** 10.3389/frhs.2025.1477530

**Published:** 2025-10-30

**Authors:** John R. Litaker, Carlos Lopez Bray, Naomi Tamez, Wesley Durkalski, Richard Taylor

**Affiliations:** ^1^ Health Scientist and Consultant, The Litaker Group, LLC, Austin, TX, United States; ^2^ Research Associate, Sendero Health Plans, Austin, TX, United States; ^3^ Chief Executive Officer Emeritus, Sendero Health Plans, Austin, TX, United States; ^4^ Clinical Assistant Professor, University of Texas at Austin, Austin, TX, United States

**Keywords:** vaccine uptake, moveable middle, public health emergency, COVID-19, Affordable Care Act, Sendero Health Plans, vaccine acceptors, vaccine refusers

## Abstract

**Background:**

Vaccine hesitancy is a multifactorial construct that posits vaccine uptake is based on person, place, time, and vaccine type. This study sought to identify individuals at about the six-month mark of COVID-19 vaccine availability in Central Texas to determine if they were vaccine acceptors, vaccine refusers, or in the moveable middle using the COVID-19 Vaccination Uptake Behavioral Science Task Force framework developed for the US Centers for Medicare and Medicaid Services and to disentangle individuals in the moveable middle to either vaccine acceptors or vaccine refusers.

**Methods:**

An online survey was distributed to individuals with Affordable Care Act insurance to assess: (1) COVID-19 vaccine uptake; and (2) plans to obtain a COVID-19 vaccine for those who had not yet received at least one dose of a COVID-19 vaccine. The study period was June 27, 2021, through July 13, 2021. Quantitative and qualitative data were collected.

**Results:**

900 individuals participated in this study. The point prevalence of COVID-19 vaccine acceptance and refusal was 94.9% (*n* = 854) and 5.1% (*n* = 46), respectively. For those who were initially identified in the moveable middle, 84.6% exited the moveable middle as vaccine refusers. Black or African American race (*p* < 0.001), income level (*p* = 0.004), and education level (*p* = 0.015) were associated with obtaining at least one dose of the COVID-19 vaccine.

**Conclusions:**

Real-world evidence at the time of a public health emergency can be used to determine point prevalence of vaccine uptake to stratify individuals as vaccine acceptors, vaccine refusers, or the moveable middle. Such evidence can be used to support health policy and planning during a public health emergency.

## Background

Vaccine hesitancy is a multifactorial construct that posits vaccine uptake is based on person, place, time, and vaccine type (1). A person who is vaccine hesitant may refuse one or more vaccines, accept a vaccine despite ongoing concerns, or delay obtaining a vaccine until specific conditions are met (2). Descriptions of vaccine hesitancy typically focus on predictive factors of vaccine acceptance or refusal, including sociodemographic data and qualitative constructs (3, 4). Research on COVID-19 vaccine hesitancy has revealed that women, individuals with less formal education, people with lower household income, and individuals who identify as Black or African American are less likely to obtain the COVID-19 vaccine (3, 5–8). Beyond COVID-19, vaccine hesitancy has also been associated with smallpox, the 1976 swine flu, and the diphtheria, tetanus, and pertussis vaccines (9).

The COVID-19 Vaccination Uptake Behavioral Science Task Force for the US Centers for Medicare and Medicaid Services developed a framework to assess vaccine hesitancy among employees of long-term care facilities (10). Brieﬂy, this framework stratiﬁed long-term care facility employees into three vaccine uptake categories: (1) vaccine acceptors; (2) vaccine refusers; and (3) the moveable middle. Vaccine acceptors are people who have agreed to receive a vaccine and can potentially act as positive inﬂuencers and ambassadors to those who have not yet received the vaccine. Vaccine refusers are people who have indicated that they will not receive a vaccine and can potentially act as negative inﬂuencers to those who may be undecided. The moveable middle are people who may become vaccine acceptors, refusers, or remain undecided.

A key tenet of the moveable middle is that these individuals, while currently unvaccinated and hesitant to receive the vaccine, are at some point likely to exit the moveable middle to become either a vaccine acceptor or a vaccine refuser. It is postulated that individuals in the moveable middle can be encouraged to become vaccine acceptors if logistical and access barriers can be mitigated, social influence and emotions can be harnessed, and if trust in vaccine safety is built by using authentic peer-to-peer conversations (10). Otherwise, individuals may exit the moveable middle to become vaccine refusers. Currently, there is a paucity of information about the direction a person takes when they exit the moveable middle.

The primary purpose of this study was to identify individuals at about the six-month mark of COVID-19 vaccine availability to determine if they were vaccine acceptors, vaccine refusers, or in the moveable middle. For individuals in the moveable middle, our secondary purpose was to assess whether they were likely to exit the moveable middle as vaccine acceptors or vaccine refusers. Finally, we sought to determine the point prevalence of vaccine acceptors and refusers after six months of COVID-19 vaccine availability once we applied the Task Force framework and disentangled individuals from the moveable middle into either the vaccine acceptor or vaccine refuser category.

## Methods

Eligible participants for this study were Sendero head-of-household members. Head-of-household members are deﬁned as adult members 18 years old or older who are the primary policyholder for a Sendero health insurance plan. Sendero is a taxpayer-supported health insurance company offering health insurance in Central Texas as part of the Affordable Care Act. Sendero distributed an online survey to eligible participants to assess: (1) COVID-19 vaccine uptake among members; and (2) plans to obtain a COVID-19 vaccine for members who have not yet received at least 1 dose of a COVID-19 vaccine. Individuals were invited to participate either by email or by post, depending on the communication preference previously expressed by the member. All individuals had a minimum of two weeks to complete the survey. The study period was June 27, 2021, through July 13, 2021. Staged COVID-19 vaccine distribution was initiated in Central Texas in late December 2020 and made freely available to all adults by June 2021. No US Centers for Disease Control and Prevention (CDC) priority restrictions related to vaccine availability were in place at the time this survey was conducted. By the time this survey was distributed, all recipients would have had a six month opportunity to obtain at least one dose of the COVID-19 vaccine. Indeed, excess vaccination capacity began emerging in late March 2021 in Austin, Texas (i.e., there were more appointment slots available than being filled beginning at this time).

All responses were submitted using the online Qualtrics platform (Qualtrics, Provo, UT, USA). Participation in the survey was voluntary, and those who completed the survey were sent a USD $25.00 gift card to a local grocery merchant. All communication was provided in English and Spanish. All data were de-identiﬁed prior to analysis. Pairwise deletion was used to address cases of missing data.

Variables of interest included sociodemographic factors such as age, sex at birth, race, ethnicity, level of educational attainment, and income. Age in years was computed using the difference between the survey completion date and the member's date of birth. All statements or questions, except those associated with race and ethnicity, required a single response. The survey allowed individuals to self-identify with multiple racial or ethnic identities to reﬂect the diversity of respondents' racial and ethnic heritage. Descriptions of univariate categorical variables include count and percent for each level of the variable, and quantitative variables include frequency, percent, mean, and standard deviation. Qualitative feedback is reported verbatim based on member input unless otherwise specified, except that minor spelling errors were corrected. To conserve power, for analyses performed among subsets of respondents, we chose to dichotomize selected demographic and social determinant of health variables. As such, race categories were subsequently dichotomized to include a single race category vs. all other races. Ethnicity was subsequently dichotomized to Hispanic, Latino, or Spanish origin vs. not Hispanic, Latino, or Spanish origin. Education was subsequently dichotomized to achieved at least a bachelor degree vs. achieved less than a bachelor degree.

The outcome variables of interest include whether a respondent had obtained the COVID-19 vaccine or planned to obtain the COVID-19 vaccine, as well as associated qualitative data. The following survey questions were relevant to the outcome variables of interest:

1. Have you received at least one dose of the COVID-19 vaccine? Response options were *Yes* or *No*.
2. If No to (1), do you plan to get the COVID-19 vaccine? Response options were *Yes*, *No*, *Not sure*, or *Prefer not to answer*. All individuals who provided responses other than *Yes* for question 2 were also asked Questions 3 and 4.
3. Please tell us why you answered [*No*, *Not sure*, or *Prefer not to answer*] in [Question 2]. Open text responses with unlimited characters were used to record answers.
4. What, if anything, could be done to change your mind from [*No*, *Not sure*, *Prefer not to answer*] to “Yes, I plan to get the COVID-19 vaccine?” Open text responses with unlimited characters were used to record answers.

Responses to these four questions were restructured as follows.

• A person was deemed to be a **vaccine acceptor** if they answered *Yes* to question 1 (“Have you received at least one dose of the COVID-19 vaccine?” or if they answered *Yes* to question 2 (“Do you plan to get the COVID-19 vaccine?”) or if they provided qualitative feedback to question 4 (“What, if anything, could be done to change your mind from *No*, *Not sure*, or *Prefer not to answer* to *Yes*, I plan to get the COVID-19 vaccine?” that indicated a plausible and likely possibility to obtain the vaccine.
• A person was deemed to be a **vaccine refuser** if they answered *No* to question 1 (“Have you received at least one dose of the COVID-19 vaccine?”) and answered *No* to Question 2 (“Do you plan to get the COVID-19 vaccine?”) and if they provided qualitative feedback to question 4 (“What, if anything, could be done to change your mind from *No* to *Yes*, I plan to get the COVID-19 vaccine?” that indicated that they would not obtain the vaccine.
• A person was deemed to be in the **moveable middle** if they answered *Not sure* or *Prefer not to answer* to question 2 (“Do you plan to get the COVID-19 vaccine?”). Individuals in the moveable middle were further reclassified as a vaccine acceptor or vaccine refuser based on qualitative feedback to question 4 (“What, if anything, could be done to change your mind from *No* to *Yes*, I plan to get the COVID-19 vaccine?”) as noted above.

Appropriate analyses for a cross-sectional survey design were used. Unadjusted bivariate analyses were performed to describe relationships between variables and to identify statistically significant independent variables for regression analysis if indicated. The chi-square test for independence (*χ*^2^) with corresponding degrees of freedom [*χ*^2^ (df)] were used to describe associations between categorical independent and dependent variables, and corresponding *P*-values (*p*) are reported. Unadjusted bivariate analyses assume the null form of no association between the variables. The *a priori* type I error rate was set to alpha = 0.05.

## Results

Of the 5,806 members invited to participate in this study, 900 (15.5%) submitted a complete survey. The response rate is consistent with head-of-household survey response rates for other Sendero population health research initiatives (3, 11). Females represented 50.6% (*n* = 455) of respondents. The average age of respondents was 47.8 years, with a standard deviation of ±12.1 years. Individuals who identified as White represented 745 (79.4%) respondents, while individuals who self-identified as Asian and Black or African American represented 54 (5.8%) and 45 (4.8%) respondents, respectively. About one-fifth of respondents (20.9%; *n* = 188) self-identified as Hispanic, Latino, or of Spanish origin. The majority of respondents (53.1%; *n* = 478) reported having obtained at least a bachelor degree. Eleven percent (*n* = 99) reported obtaining a high school degree or equivalent. Of the 900 respondents, 781 (86.8%) provided information on their annual household income range, the majority of whom reported a household income of less than USD $40,000 (56.2%; *n* = 439). Table 1 reports the demographic and summary characteristics of survey respondents.

**Table 1:** Reported demographic and summary characteristics of survey respondents.

Respondent demographics	Total cohort *N* (%)	Received at least one dose of the COVID-19 vaccine *N* (%)	Did not receive a dose of the COVID-19 vaccine *N* (%)
Sex	*N* = 900	827 (91.9)	73 (8.1)
Female	455 (50.6)	414 (50.1)	41 (56.2)
Male	445 (49.4)	413 (49.9)	32 (43.8)
Age in Years (Range: 20–86 years) 47.80 ± 12.1 years	*N* = 900	827 (91.9)	73 (8.1)
18–24 years old	16 (1.8)	16 (1.9)	0 (0)
25–34 years old	142 (15.8)	131 (15.8)	11 (15.1)
35–44 years old	209 (23.2)	187 (22.6)	22 (30.1)
45–54 years old	213 (23.7)	195 (23.6)	18 (24.7)
55–64 years old	306 (34)	285 (34.5)	21 (28.8)
≥ 65 years old	14 (1.6)	13 (1.6)	1 (1.4)
Race^a^	*N* = 938^a^	860 (91.7)	78 (8.3)
American Indian or Alaskan Native	14 (1.5)	12 (1.4)	2 (2.6)
Asian	54 (5.8)	52 (6.0)	2 (2.6)
Black or African American	45 (4.8)	33 (3.8)	12 (15.4)
Native Hawaiian or Other Pacific Islander	4 (0.4)	4 (0.5)	0 (0.0)
White	745 (79.4)	691 (80.3)	54 (69.2)
Other	76 (8.1)	68 (7.9)	8 (10.3)
Ethnicity^a^	*N* = 900	827 (91.9%)	73 (8.1)
No, not of Hispanic, Latino, or Spanish origin	712 (79.1)	660 (79.8)	52 (71.2)
Yes, Hispanic, Latino, or Spanish origin (More than one ethnicity could be selected)	188 (20.9)	167 (20.2)	21 (28.8)
Mexican, Mexican American, Chicano	124 (66.0)	110 (65.9)	14 (66.7)
Puerto Rican	3 (1.6)	3 (1.8)	0 (0)
Cuban	7 (3.7)	5 (3.0)	2 (9.5)
Another Hispanic, Latino, or Spanish origin	59 (31.4)	53 (31.7)	6 (28.6)
Education	*N* = 900	827 (91.9)	73 (8.1)
Less than high school	6 (0.7)	6 (0.7)	0 (0)
Some high school	37 (4.1)	31 (3.7)	6 (8.22)
High School Diploma, GED, or equivalent	99 (11)	86 (10.4)	13 (17.8)
Some College	198 (22)	179 (21.6)	19 (26.0)
Trade School	5 (0.6)	5 (0.6)	0 (0)
Associate Degree	74 (8.2)	65 (7.9)	9 (12.3)
Bachelor's Degree	323 (35.9)	307 (37.1)	16 (21.9)
Master's Degree	154 (17.1)	145 (17.5)	9 (12.3)
Doctorate	1 (0.1)	1 (0.1)	0 (0)
Other	3 (0.3)	2 (0.2)	1 (1.4)
Annual Household Income	*N* = 900	827 (91.9)	73 (8.1)
Less than $10,000 per year	68 (7.6)	56 (6.8)	12 (16.4)
$10,000–$29,999	261 (29)	241 (29.1)	20 (27.4)
$30,000–$39,999	110 (12.2)	98 (11.9)	12 (16.4)
$40,000–$49,999	95 (10.6)	92 (11.1)	3 (4.1)
$50,000–$75,999	113 (12.6)	105 (12.7)	8 (11.0)
$76,000–$99,999	59 (6.6)	57 (6.9)	2 (2.7)
$100,000 or above	75 (8.3)	71 (8.6)	4 (5.5)
Other	7 (0.8)	6 (0.7)	1 (1.4)
Prefer Not To Answer	112 (12.4)	101 (12.2)	11 (15.1)

^a^ Respondents were able to represent their racial and ethnic heritage by selecting more than one racial or ethnic group.

A total of 827 (91.9%) respondents indicated that they had received at least one dose of a COVID-19 vaccine; with about half (49.8%, *n* = 412) receiving the Moderna vaccine, 41.6% (*n* = 344) receiving the Pfizer vaccine, and 7.5% (*n* = 62) receiving the Johnson & Johnson vaccine. The remaining 1.1% (*n* = 9) could not recall which vaccine they received. A total of 73 respondents indicated they had not received at least one dose of a COVID-19 vaccine. For those who had not obtained the vaccine 22 (30.1%), 25 (34.2%), 24 (32.9%), and 2 (2.7%) indicated *Yes*, *No*, *Not sure*, and *Prefer not to answer*, respectively to the questions of whether they planned to receive the COVID-19 vaccine.

These 73 respondents were further categorized as vaccine acceptors, vaccine refusers, or in the moveable middle. Table 2 summarizes qualitative feedback from the 73 respondents who indicated that they had not received at least one dose of the COVID-19 vaccine, why they chose this response, and what if anything could be done to change their mind and obtain the vaccine. Respondents were further classified as vaccine acceptors or vaccine refusers based on their feedback. The 22 persons who indicated that they planned to obtain the COVID-19 vaccine were deemed vaccine acceptors. The 25 persons who said they did not plan to obtain the COVID-19 vaccine were deemed vaccine refusers, with one person recategorized as a vaccine acceptor based on further review of qualitative data. The 24 and two persons who were *Not sure* or who *Preferred not to answer*, respectively, were deemed to be in the moveable middle. These 26 persons were further assessed based on qualitative feedback to determine if there was anything that could be done to change their minds about obtaining the COVID-19 vaccine. Four persons in the moveable middle indicated a plausible scenario that would cause them to change their minds and become vaccine acceptors, while 22 people indicated a scenario that would have them become vaccine refusers.

**Table 2:** Qualitative feedback from members who had not obtained at least one dose of a COVID-19 vaccine, their plans to obtain the COVID-19 vaccine, and classification of their feedback as either accepting or refusing to obtain the COVID-19 vaccine (*n* = 73).

If No to (1), do you plan to get the COVID-19 vaccine?	Please tell us why you answered [*No*, *Not sure*, or *Prefer not to answer*] in [Question 2]	What, if anything, could be done to change your mind from [*No*, *Not sure*, *Prefer not to answer*] to “Yes, I plan to get the COVID-19 vaccine?”	Classification as vaccine acceptor or vaccine refuser
Not sure	Scared of possible reactions even though I know they are slim and I know it would protect me from the virus. I would hate to be one of those one in a million but I guess it goes both ways.	To speak to a doctor	Vaccine acceptor
Not sure	Want my Doctor's advice on it and which of the shots he feels is the best one.	What my Doctor recommends.	Vaccine acceptor
No	Not interested, healthy and active adult.	Make it mandatory for travel.	Vaccine acceptor
Not sure	I'm just not sure	If I have to get it for a job or to travel	Vaccine acceptor
Not sure	I will get one if necessary for employment reasons	Employer mandate	Vaccine acceptor
Not sure	I work from home and only leave to get groceries. I also know of people who have gotten a high fever after receiving the shot and I have many maladies that make even getting a cold something of worry. So I'm a little leery of it.	Time, in all likelihood.	Vaccine refuser
Not sure	Would like more data	time	Vaccine refuser
Not sure	I am still thinking about it	n/a	Vaccine refuser
Not sure	…	…	Vaccine refuser
Not sure	Just not sure. It needs to be tested a little more to me.	Not sure yet.	Vaccine refuser
Not sure	Because of my experience with the flu vaccine and because the covid 19 vaccine came out too soon, I'm reluctant to take it.	I don't know.	Vaccine refuser
Not sure	I feel immense social, political and travel pressure to obtain this medical treatment. I believe in the right to choose what I put into my body. I am concerned about the long term and short term effects of the vaccine vs. the possibility of getting covid and the effects of that. Seems like choosing between two horrible choices.	1. More years of research as to the efficacy, side effects and long term health ramifications of receiving an “experimental” (emergency only FDA approved) drug. 2. Right to privacy concerning receiving vaccination. It is a drug, private health information and I do not believe we should be asking, telling or questioning people on whether they received a medical treatment. It's an invasion of privacy. 3. More time, more data on side effects especially as it relates to auto immune diseases such as Hashimoto's or other endocrine disorders.	Vaccine refuser
Not sure	Autoimmune	More data regarding long term safety	Vaccine refuser
Not sure	Don't trust that it has been handled correctly.	More data over time to show no side effects and that they will perform antibody test to show that it did actually work.	Vaccine refuser
Not sure	I did not ever get COVID. Complete information not provided on shot. Worried about side effects. Who and why did people die from COVID and some lived?	More information and explanation on why some got COVID and some didn't. Some died some didn't.	Vaccine refuser
Not sure	I am going to wait a while to make sure that they know what any side effects or complications are, they brought this drug to the market pretty fast. My husband has already had Covid and I lived in very close quarters with him without any precautions. We are both healthy and don't have any conditions that makes covid worse.	My husband has already had Covid and I lived in very close quarters (800sf) with him without any precautions. We are both healthy and don't have any conditions that makes covid worse, so I am going to wait.	Vaccine refuser
Not sure	Just haven't felt the necessity at this point. But not opposed.	More education in why it is vital.	Vaccine refuser
Not sure	I wanna learn more about it before making a decision	Let me know that I won't get COVID if I do get the shots.	Vaccine refuser
Not sure	Still hearing mixed reviews and side effects	Not sure what could really change my mind. Keep hearing side effects.	Vaccine refuser
Not sure	Por mi enfermedad no me siento segura de recibir la vacuna contra el covid 19	Nada	Vaccine refuser
Not sure	Bad reactions to vaccines	None	Vaccine refuser
Not sure	Porque sigo proteguiendome y sigo esperando mas informacion de las vacunas y sus efectos secundarios	Nada, yo sabre el momento mas seguro para aserlo	Vaccine refuser
Not sure	My health is not good	Nothing	Vaccine refuser
Not sure	Porque no están aprobadas fda	Nada	Vaccine refuser
Not sure	Like I said before it's too much up and down about it it's a lot of things that's not being told and said about it so right now if the Lord don't put it up on my heart to get it I'm not worrying about it if enough people in the world get it I think I'll be okay	At the moment there is nothing that I can think of that's going to change my mind like I said when the Lord put up on my heart I'll do it just like I did the flu shot	Vaccine refuser
No	I want to see the long term effects on humans before getting it.	Nothing immediate. Time will tell whether I feel the vaccine is safe and effective or not.	Vaccine refuser
No	No answer	No answer	Vaccine refuser
No	I don't trust big Pharma.	Allow the public to openly discuss all vaccines and vaccine effects on all social media. Have our Government tell us the truth.	Vaccine refuser
No	Phase I, II and III were run concurrently and Phase I had a whopping 45 people tested (Moderna) with Phase III years out from completion. Novel technology never approved for human use. 99+% false positives on Covid testing when prevalence is low, due to the nature of PCR testing and statistics for all diagnostic tests (even stated by FDA and known by those in the industry). Did not agree with FDA's risk-benefit analysis when personally reviewing what was submitted to allow use of the vaccines without the normal clinical trial process. I consider this all a large scale, uniformed consent clinical trial.	Completed clinical trials, but since the control group is now compromised, probably never. Would consider a vaccine that underwent a “normal” study of dead virus. Oh wait, Covid-19 isolation is not necessarily being used. Oh wait, we'll never know the true prevalence of this disease, etc.	Vaccine refuser
No	I don't trust the safeness of it nor the government push for it.	Years of testing for safety and open exposure of all side effects.	Vaccine refuser
No	It is NOT been properly tested … and do not trust it	nothing but better testing, and not on humans	Vaccine refuser
No	Hasn't had favorable reviews, hasn't been out long enough to know all side effects	Out long enough to know all side effects	Vaccine refuser
No	I am not at risk and prefer not to put things in my body, that “may” cause side effects.	I could get older and the chances of having adverse reactions to Covid were higher … or give me 100% guarantee of no side effects. But as it stands, I think taking the shot is just as risky as getting Covid for me. And I'd rather just be cautious about Covid.	Vaccine refuser
No	It is a waste of time. I have no risk of serious danger if I become infected with the virus.	I might consider it a little more if it was actually something to be scared of.	Vaccine refuser
No	More accurate would be “not yet”.	Fully tested and approved, then maybe	Vaccine refuser
No	Regarding mRNA gene therapy: even if these are fully approved by the FDA, I will not voluntarily undergo this treatment for at least one decade. I appreciate the urgency of the treatment, but I am not willing to risk my health for an experimental procedure. Regarding the J&J Vaccine: I would consider getting a viral vector vaccine but I don't J&J's ethical integrity	If a traditional viral vector vaccine, produced by a pharmaceutical company with a history of responsible practices, were to be fully approved by the FDA and distributed in the United States, I would likely get that vaccine	Vaccine refuser
No	Don't feel it is necessary	NOTHING	Vaccine refuser
No	For the same reason I don't get the flu vaccine. I would rather take precautions like wearing a mask, social distancing and avoid risky situations than to take a chance with the vaccine. I am hyper sensitive to medications and avoid them whenever possible. I am a healthy individual without underlying health conditions. I believe my risk of being seriously ill due to covid is extremely minimal.	At this time, nothing will change my mind. I prefer to continue living as I did prior to a vaccine being available. I protect myself and others by wearing a mask, social distancing and avoiding riskier behaviors.	Vaccine refuser
No	This is not an approved vaccine, survival rate Is over 99%. Me and my son had covid back in August of 2,020 don't find thats it needed,	Nothing don't trust the whole thing, when there are drugs that they could give you to help with the virus, but because of big pharm who want you to take the vaccine, get real	Vaccine refuser
No	I do not trust the pharmaceutical industry, or the government. Nor really western medicine generally. I wish insurance covered more alternative, natural health-promoting practices. Or even just sensible ones, like ivermectin is effective against this virus. But bc they can't make a massive profit off it, it's suppressed. If you really cared about your clients, you would provide them all with this prophylactic and lifesaving treatment. Not pushing dangerous expensive ventilators and such.	Nothing. Also now I can't get it anyways because I'm getting pregnant.	Vaccine refuser
No	I have multiple, serious allergies and people with multiple or severe allergic reactions are advised not to get the shot.	Nothing at this point.	Vaccine refuser
No	What's in it. It's not a vaccine it's a shot it's not a cure for Covid-19 that's my opinion	No !	Vaccine refuser
No	I don't trust it and doctors	Nothing	Vaccine refuser
No	Personal	Nothing	Vaccine refuser
No	I am not personally worried about coronavirus, and there are risks with the vaccines.	Nothing.	Vaccine refuser
No	Don't want to	Nothing	Vaccine refuser
No	My personal belief	Nothing	Vaccine refuser
No	I already had it, did not get sick and have extraordinarily high antibody count after 100 days	None	Vaccine refuser
No	Don't want it	No	Vaccine refuser
PNTS	Privacy of health information	Prefer not to answer	Vaccine refuser
PNTS	I prefer not to answer	Not sure	Vaccine refuser

Selected demographic variables and social determinants of health were associated with receiving at least one dose of the COVID-19 vaccine (the dependent variable). Evidence from this study indicates an association for individuals who included Black or African American in their self-reported race profile [*χ*^2^(1) = 15.83, *p*-value <.001], education level [*χ*^2^(2) = 11.33, *p*-value = .004], and annual household income [*χ*^2^(6) = 15.79, *p*-value = .015] and receiving at least one dose of COVID-19 vaccine, respectively (see Table 3). There were 73 study participants who indicated they had not received at least one dose of COVID-19 vaccine (Table 4). Subsequent qualitative responses to the follow-up statement, “I plan to obtain the COVID-19 vaccine” were categorized into three categories, *acceptor*, *moveable middle*, or *refuser* (an outcome variable of interest). Analysis of selected demographic and social determinants of health (SDoH) variables for associations with categorized responses for “I plan to obtain a COVID-19 vaccine,” identified statistical associations between ethnicity [*χ*^2^(2) = 8.69, *p*-value = .013], which included Hispanic, Latino or Spanish origin vs. Not of Hispanic, Latino, or Spanish origin, and education level [*χ*^2^(2) = 7.92, *p*-value = .019], which compared participants who obtained at least a bachelor degree vs. less than a bachelor degree, respectively.

**Table 3:** Results of chi-square tests of independence for based on initial COVID-19 vaccination status.

Variable	Total Cohort *N* (%)	I received at least one dose of the COVID-19 vaccine		Chi-square (df), *P* value
		Yes	No	
		*N* (%)	*N* (%)	
Sex	900	827 (91.9)	73 (8.1)	
Female	455 (50.6)	414 (50.1)	41 (56.2)	.77 (1), *p* = .380
Male	445 (49.4)	413 (49.9)	32 (43.8)	
Age Level	900	827 (91.9)	73 (8.1)	
18–34 years of age	158 (17.6)	147 (17.8)	11 (15.1)	2.14 (2), *p* = .343
35–49 years of age	312 (34.7)	281 (34)	31 (42.5)	
50+ year of age	430 (47.8)	399 (48.2)	31 (42.5)	
Race^a^	938	860 (91.7)	78 (8.3)	
American Indian or Alaskan Native	14 (1.5)	12 (1.4)	2 (2.6)	0.12 (1), *p* = .729^e^
Asian	54 (5.8)	52 (6.0)	2 (2.6)	.83 (1), *p* = .362^e^
Black or African American	45 (4.8)	33 (3.8)	12 (15.4)	15.83 (1), *p* < .001^e^
Native Hawaiian or Other Pacific Islander	4 (0.4)	4 (0.5)	0 (0.0)	n/a
White	745 (79.4)	691 (80.3)	54 (69.2)	0.31 (1), *p* = .578^e^
Other	76 (8.1)	68 (7.9)	8 (10.3)	0.27 (1), *p* = .603^e^
Ethnicity^a^	900	827 (91.9)	73 (8.1)	
Not of Hispanic, Latino, or Spanish origin	712 (79.1)	660 (79.8)	52 (71.2)	2.49 (1), *p* = .115^e^
Hispanic, Latino, or Spanish origin	188 (20.9)	167 (20.2)	21 (28.8)	
Mexican, Mexican American, Chicano	124 (64.2)	110 (57.0)	14 (63.6)	1.49 (1), *p* = .222
Puerto Rican	3 (1.6)	3 (1.6)	0 (0.0)	n/a
Cuban	7 (3.6)	5 (2.6)	2 (9.1)	n/a
Other Hispanic, Latino, or Spanish origin	59 (30.6)	53 (27.5)	6 (27.3)	n/a
Education Level	897	825 (92.0)	72 (8.0)	
≤ High School Diploma^b^	43 (4.8)	37 (4.5)	6 (8.2)	11.33 (2), *p* = .004
≥ HS Diploma < Bachelor's Degree^c^	376 (41.9)	335 (40.5)	41 (56.2)	
≥ Bachelor's Degree^d^	478 (53.3)	453 (54.8)	25 (34.2)	
Annual Household Income	781	720 (92.2)	61 (7.8)	
<$10,000	68 (0.1)	56 (7.8)	12 (19.7)	15.79 (6), *p* = .015
$10,000–$29,999	261 (0.3)	241 (33.5)	20 (32.8)	
$30,000–$39,999	110 (0.1)	98 (13.6)	12 (19.7)	
$40,000–$49,999	95 (0.1)	92 (12.8)	3 (4.9)	
$50,000–$75,999	113 (0.1)	105 (14.6)	8 (13.1)	
$76,000–$99,999	59 (0.1)	57 (7.9)	2 (3.3)	
$100,000 or more	75 (0.1)	71 (9.9)	4 (6.6)	

^a^ Respondents were able to represent their racial and ethnic heritage by selecting more than one racial or ethnic group.

^b^ Includes respondents who self-identified as attaining an education level of “Less than some high school,” “Some high school,” and “High School Diploma, GED, or equivalent”.

^c^ Includes respondents who self-identified as attaining an education level of “Trade school,” “Some college,” and “Associate degree”.

^d^ Includes respondents who self-identified as attaining an education level of “Bachelor Degree” or “Graduate Degree.”.

n/a: Cannot calculate due to small cell size.

^e^ These samples are independent and based on the appropriate independent sample denominator for the calculation of interest.

**Table 4:** Results of chi-square tests of independence for individuals who had not received a dose of the COVID-19 vaccine and were further classified as vaccine acceptors, the moveable middle, or vaccine refusers based on a response to the question “Do you plan to obtain the COVID-19 vaccine?”.

Variable	*N* (%)	Respondents who did not receive at least one dose of the COVID-19 vaccine were asked to respond to the following statement: “I plan to obtain the COVID-19 vaccine” Responses were classified as acceptors, the moveable middle, or refusers			Chi-square (df), *P* value
		Acceptor	Moveable Middle	Refusers	
		*n* (%)	*n* (%)	*n* (%)	
Sex	73	22 (30.1)	26 (35.6)	25 (34.2)	
Female	41 (56.2)	12 (54.5)	15 (57.7)	14 (56.0)	0.05 (2), *p* = .976
Male	32 (43.8)	10 (45.5)	11 (42.3)	11 (44.0)	
Age Level	73	22 (30.1)	26 (35.6)	25 (34.2)	
18–34 years of age	11 (15.1)	5 (22.7)	4 (15.4)	2 (8.0)	3.19 (4), *p* = .527
35–49 years of age	31 (42.5)	9 (40.9)	9 (34.6)	13 (52.0)	
50+ year of age	31 (42.5)	8 (36.4)	13 (50.0)	10 (40.0)	
Race^a^	78^a^	25 (32.5)	27 (35.1)	26 (33.8)	
American Indian or Alaskan Native	2 (2.6)	1 (4.0)	0 (0)	1 (3.8)	n/a
Asian	2 (2.6)	1 (4.0)	1 (3.7)	0 (0)	n/a
Black or African American	12 (15.4)	4 (16.0)	3 (11.1)	5 (19.2)	n/a
Native Hawaiian or Other Pacific Islander	0 (0)	0 (0)	0 (0)	0 (0)	n/a
White	54 (69.2)	18 (72.0)	17 (63.0)	19 (73.1)	0.14 (2), *p* = .931^e^
Other	8 (10.3)	1 (4.0)	6 (22.2)	1 (3.8)	n/a
Ethnicity^a^	73^a^	22 (29.7)	26 (43.2)	25 (25.7)	
Not of Hispanic, Latino, or Spanish origin	52 (71.2)	12 (54.5)	17 (65.4)	23 (92.0)	8.69 (2), *p* = .013^e^
Hispanic, Latino, or Spanish origin	21 (28.8)	10 (45.5)	9 (34.6)	2 (8.0)	
Mexican, Mexican American, Chicano	14 (63.6)	6 (60.0)	6 (66.7)	2 (100)	n/a
Puerto Rican	0 (0)	0 (0)	0 (0)	0 (0)	n/a
Cuban	2 (9.5)	1 (10.0)	1 (11.1)	0 (0)	n/a
Other Hispanic, Latino, or Spanish origin	6 (42.9)	3 (30.0)	3 (33.3)	0 (0)	n/a
Education Level	72	22 (30.1)	25 (35.6)	25 (34.2)	
≤ High School Diploma^b^	6 (8.3)	3 (13.6)	3 (12.0)	0 (0)	n/a
≥ HS Diploma < Bachelor's Degree^c^	41 (56.9)	13 (59.1)	17 (68.0)	11 (44.0)	
≥Bachelor's Degree^d^	25 (34.7)	6 (27.3)	5 (20.0)	14 (56.0)	7.92 (2), *p* = .019
Annual Household Income (USD)	61	17 (27.9)	23 (37.7)	21 (34.4)	
<$10,000	12 (19.7)	6 (35.3)	4 (17.4)	2 (9.5)	
$10,000–$29,999	20 (32.8)	6 (35.3)	8 (34.8)	6 (28.6)	
$30,000–$39,999	12 (19.7)	1 (5.9)	6 (26.1)	5 (23.8)	
$40,000–$49,999	3 (4.9)	2 (11.8)	1 (4.3)	0 (0)	1.68 (2), *p* = .431
$50,000–$75,999	8 (13.1)	1 (5.9)	3 (13.0)	4 (19.0)	
$76,000–$99,999	2 (3.3)	0 (0)	1 (4.3)	1 (4.8)	
$100,000 or more	4 (6.6)	1 (5.9)	0 (0)	3 (14.3)	

^a^ Respondents were able to represent their racial and ethnic heritage by selecting more than one racial or ethnic group.

^b^ Includes respondents who self-identified as attaining an education level of “Less than some high school,” “Some high school,” and “High School Diploma, GED, or equivalent.”.

^c^ Includes respondents who self-identified as attaining an education level of “Trade school,” “Some college,” and “Associate degree”.

^d^ Includes respondents who self-identified as attaining an education level of “Bachelor Degree” or “Graduate Degree.”.

n/a: Cannot calculate due to small cell size.

^e^ These samples are independent and based on the appropriate independent sample denominator for the calculation of interest.

**Figure 1:**
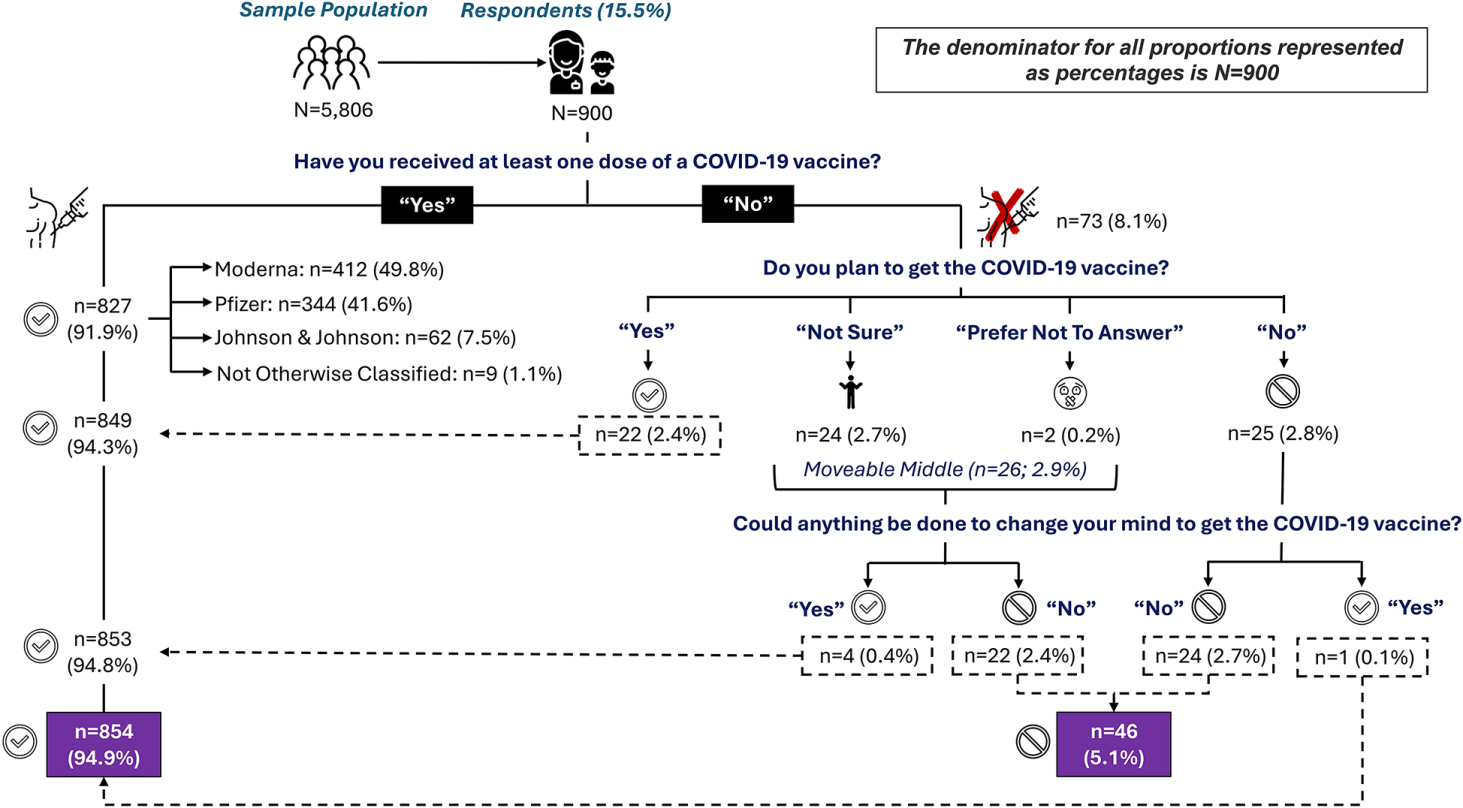
Distribution of responses regarding COVID-19 uptake six months post vaccine availability in Central Texas.

At the time of the survey 827 respondents (91.9%) indicated they had received at least one dose of COVID-19 vaccine while 73 respondents (8.1%) had not received at least one dose of the COVID-19 vaccine. Further questioning of survey respondents provided additional information to categorize respondents into either vaccine acceptors or vaccine refusers. Ultimately, 854 (94.9%) were categorized as vaccine acceptors, while 46 (5.1%) were categorized as definitive vaccine refusers (as described in Figure 1).

## Discussion

To our knowledge, this is the first study to assess the COVID-19 Vaccination Uptake Behavioral Science Task Force model of vaccine acceptors, vaccine refusers, and the moveable middle focusing on the exit strategy of individuals from the moveable middle to become either vaccine acceptors or vaccine refusers. The paucity of data on the moveable middle, and a particular paucity of data with regard to disentangling the moveable middle into its constituent parts, is a current gap in the literature. Understanding what leads people to be in the moveable middle and their plan for eventual exit from the moveable middle is an important part of the vaccine hesitancy discussion. We discuss each of the Task Force categories below.

### Vaccine acceptors

Vaccine acceptors are individuals who have obtained the COVID-19 vaccine or who plan to obtain it. In this study, 91.9% of respondents had obtained at least one dose of a COVID-19 vaccine. Of the 73 persons who had not obtained the vaccine, 30.1% (*n* = 22) indicated they planned to get it. This increased the overall proportion of vaccine acceptance from 91.9% (*n* = 827) to 94.3% (*n* = 849). Five additional individuals indicated a willingness to obtain the COVID-19 vaccine when asked, “What, if anything, could be done to change your mind from [*No*], [*Not sure*], [*Prefer not to answer*] to ‘Yes, I plan to get the COVID-19 vaccine?’” These five individuals were originally classified as a vaccine refuser (*n* = 1) or in the moveable middle (*n* = 4) and are discussed further in their respective sections below. In total 94.9% (*n* = 854) of respondents have indicated that they have obtained the vaccine, plan to obtain the vaccine, or could reasonably be persuaded to obtain the vaccine.

The proportion of vaccine acceptors in our study is greater than that recorded in Travis County, Texas (66.2%) and in global estimates (75.2%) for obtaining at least one dose of the vaccine at about six months post vaccine availability (12, 13). We postulate that respondents to our survey, all of whom had purchased private health insurance on the Affordable Care Act (ACA) marketplace, may exhibit positive health behavior. Such behavior may represent a positive health investment “in the form of [increasing] preventive services and disinvestments in the form of [reducing] risky behaviors” (14). Studies indicate that holders of private health insurance are likely to exhibit positive health behaviors, mainly due to primary and secondary prevention.

A 2017 study assessing Behavioral Risk Factor Surveillance System data among individuals with and without health insurance showed higher adjusted prevalence ratios (aPR), 95% confidence intervals (95% CI), and *P*-values (*p*) of no tobacco use and increased physical activity among those with health insurance (aPR = 1.10; 95% CI = 1.09, 1.12; *p* < .001 and aPR = 1.08; 95% CI = 1.05, 1.11; *p* < .001, respectively) (15). Individuals with health insurance were also more likely to have an annual physical exam within the past year as compared to individuals without health insurance (74.4% vs. 43.3%, *p* < 0.001) (15). Similarly, a three-year analysis of the effects of ACA expansion on health behaviors showed an increase in check-ups (*p* = .001), pap tests (*p* = .05), mammograms (*p* = .01), and HIV tests (*p* = .001) for individuals with ACA coverage (14).

Specific to vaccines, Medicare recipients in the United States showed an increase in annual influenza vaccine uptake for both men and women based on adjusted odds ratios (aOR = 1.62; 95% CI = 1.28, 2.06) (16) as did a study of individuals with public and private health insurance in rural Texas as compared to those without health insurance (aOR = 2.05; 95% CI = 1.00, 4.21 and aOR = 1.77; 95% CI = 1.07, 2.92, respectively) (17).

While the COVID-19 vaccine was freely available to all, health insurance holders may exhibit positive health-seeking behaviors, irrespective of cost. Courtemanche et al. notes that generally, within the ACA marketplace, “both types of behaviors [increasing preventive behavior and reducing risky behavior] could theoretically be influenced by both the reduction in effective prices of medical services after obtaining insurance coverage and *ex ante* moral hazard from the expectation of lower out-of-pocket costs in the future if a preventable illness occurs.” (14). With regard to COVID-19 vaccination, an increased uptake of services free at the point of delivery (e.g., COVID-19 vaccination) may create an expectation of future cost savings due to limited or no illness associated with a vaccine-preventable disease, thus encouraging COVID-19 vaccine uptake among those with health insurance.

We also postulate that shifting attitudes over time may favor vaccination. For example, over the six-month period since vaccines were first introduced, individuals may have felt more comfortable with the safety of the COVID-19 vaccine if vaccinated friends and family did not suffer undue side effects. Similarly, individuals may have been vaccinated out of necessity in order to work, travel, or interact socially with friends and family. One study indicates that individuals who were unvaccinated in June 2021 and who became vaccinated in October 2021 despite having no intention to receive the vaccine did so because of work-related mandates and because of beliefs in the ability of vaccines to protect others (18).

### Vaccine refusers

Vaccine refusers are individuals who have not obtained the COVID-19 vaccine, do not plan to obtain the COVID-19 vaccine, and cannot be persuaded to obtain the COVID-19 vaccine. In this study, 2.8% (*n* = 25) of respondents had not obtained the vaccine and did not plan to obtain the vaccine. On further analysis, one of these individuals indicated a likely possibility to obtain the vaccine and was reclassified as a vaccine acceptor. An additional 22 persons exited the moveable middle (discussed in the next session) and are deemed to be vaccine refusers. In total, the proportion of vaccine refusers increased from 2.7% (*n* = 24) to 5.1% (*n* = 46) when all analyses were completed.

Vaccine refusers are thought to represent about 2%–3% of the population (11). However, real-world evidence, as demonstrated in our study, which follows individuals through a process to determine their actual plan of action, is scarce. Other studies have examined the idea of COVID-19 vaccine refusal, with 19.1% of healthcare workers in Montréal, Québec refusing the vaccine (19) and 5.3% and 9.4% of healthcare workers in California refusing or hesitant to obtain the vaccine, respectively (20). Further analysis of the Canadian findings indicates that of those who refused, 74.8% (*n* = 391) may accept the vaccine in the future (19). Therefore, a more accurate proportion of those refusing the vaccine in the Canadian cohort is 5.0% (*n* = 137), which is similar to the findings in our study.

The 25 persons who did not plan to obtain the vaccine (including the one person who was later deemed a vaccine acceptor) provided the following reasons:

• Concerns about the clinical trial process
  ○ “Phase I, II and III were run concurrently and Phase I had a whopping 45 people tested (Moderna) with Phase III years out from completion.”
  ○ “It [has] NOT been properly tested … and do not trust it.”
• Lack of trust
  ○ “I don't trust the safeness of it nor the government push for it.”
  ○ “I don't trust big Pharma.”
  ○ “I don't trust it and doctors.”
• Concerns about the long-term impact of the vaccine
  ○ “I want to see the long term effects on humans before getting it.”
• Not at risk of getting COVID-19
  ○ “I am not at risk and prefer not to put things in my body, that ‘may’ cause side effects.”
  ○ “I am not personally worried about coronavirus, and there are risks with the vaccines.”
  ○ “I already had [COVID], did not get sick and have [an] extraordinarily high antibody count after 100 days.”
  ○ “It is a waste of time. I have no risk of serious danger if I become infected with the virus.”

These same 25 persons provided additional feedback when asked, “What, if anything, could be done to change your mind from [*No*] to ‘Yes, I plan to get the COVID-19 vaccine?’” One person indicated that they could be persuaded to take the vaccine based on the response of “make it mandatory for travel.” Based on this response, we believe that given the right situation and circumstances, this individual would likely obtain the vaccine. Further, we do not assess this respondent's statement as being impractical or onerous, particularly considering that many countries instituted travel bans during the pandemic with limited movement only with proof of vaccination.

The remaining 24 persons (96.0%) had the following responses. Fourteen persons were emphatic in that they would not change their mind by responding with some version of nothing, none, or no! The remaining 10 persons offered a variety of conditions, all of which were deemed impractical to achieve, including:

• “Years of testing for safety and open exposure of all side effects;”
• “Out long enough to know all side effects;”
• “Fully tested and approved, then maybe;” and
• “ … Give me 100% guarantee of no side effects.”

This feedback echoed findings reported elsewhere, including possible side effects of the vaccines, the speed in which the vaccines were developed, lack of trust related to the science underpinning the vaccines, and a belief that the COVID-19 disease is not serious and, therefore, a vaccine is not needed (11, 19, 20).

### Moveable middle

The moveable middle includes individuals who had not obtained the COVID-19 vaccine by the time of survey administration. These individuals then responded *Not sure* or *Prefer not to answer* when asked if they planned to obtain the COVID-19 vaccine. As such, 2.9% (*n* = 26) of respondents were deemed to be in the moveable middle. When asked, “What, if anything, could be done to change your mind from [*Not sure*] [*Prefer not to answer*] to ‘Yes, I plan to get the COVID-19 vaccine?’” four (15.4%) persons indicated that they were likely to exit the moveable middle as vaccine acceptors while 24 (84.6%) persons indicated that they were likely to exit as vaccine refusers.

Individuals in the moveable middle are a heterogeneous group who ebb and flow on the vaccine hesitancy spectrum based on person, place, and time. At six months post vaccine availability, the moveable middle represented 2.9% of the overall sample in our study. We report no statistically significant differences in sociodemographic factors between the moveable middle, vaccine acceptor, and vaccine refuser groups, except for individuals who identify as Hispanic vs. no Hispanic ethnicity (*p* = .013) (Table 4). Data from this study continues a trend of decreasing movable middle prevalence previously described by our research team from 30.4% immediately prior to vaccine availability (November 11, 2020–December 21, 2020), decreasing to 16.8% in the week immediately after vaccine availability (December 24, 2020–December 31, 2020) (3, 11). Research based on the National Immunization Survey Adult COVID Module (NIS-COVID) shows similar shifting patterns among sociodemographic variables for the moveable middle over time at about six and 18 months post vaccine availability but does not report moveable middle prevalence for either time period (21). A reported decline in the percentage of US adults in the moveable middle over the study period from 26% to 3% is noted, but this finding appears to be based on CDC COVID-19 tracker data, not on NIS-COVID data (21). Regardless, the reported shift in the moveable middle to 3% at 18 months post vaccine availability mirrors our reported finding of 2.9%, albeit at six months. Another study reported approximately 24% and 26% of respondents in the United Kingdom and the Republic of Ireland, respectively, were in the moveable middle in November–December 2020 (22), which is similar, if only slightly lower, than the proportion (30.4%) we previously reported during the same period (3). While additional research is needed to better understand the decrease in prevalence among the moveable middle over time, previous research from our team indicates that improved access, advice from a physician, and building trust in vaccine safety are key components of the moveable middle that are amenable to change over time (11).

While individuals in the moveable middle are undecided in theory, the reality is quite different. Until such time that an individual obtains the vaccine, they are, in fact, *de facto* refusers. Yet, such *de facto* refusers may have less stigma associated with this decision than someone who has firmly said “no” as they retain the possibility of exiting as a vaccine acceptor (23). It is, therefore, important to disentangle the moveable middle into its constituent parts to identify who is likely to become a vaccine acceptor or refuser. Indeed, it is the condition(s) that a person attaches to his or her willingness to obtain the vaccine that acts as a deciding factor as to which category he or she will eventually occupy post-moveable middle status. In this study, respondents provided qualitative data that allowed us to consider whether a respondent was likely to exit the moveable middle as a vaccine acceptor or as a vaccine refuser (see Table 5). Of the 26 individuals in the moveable middle, four people (15.4%) indicated a condition that, if met, would allow them to exit as a vaccine acceptor. This included individuals who may obtain the vaccine once they speak to their doctor (*n* = 2) and individuals who said they would obtain the vaccine if it was mandatory for work or travel (*n* = 2). The remaining 22 (84.6%) either did not respond, said no, or indicated what we deemed to be an excessive demand that could not be practically or reasonably met and were thus deemed as vaccine refusers. For example, while one respondent indicated more education as a reason to exit as a vaccine acceptor, we deemed this person to be a vaccine refuser because it was not clear what additional education or information could be provided beyond what was currently available. Other feedback included:

• “Nothing,” “no,” “nada,” and “At the moment there is nothing that I can think of that's going to change my mind […] like I said when the Lord put up on my heart I'll do it just like I did the flu shot.”
• “More data regarding long term safety” and “More data over time to show no side effects and that they will perform antibody test to show that it did actually work.”
• “More education in why it is vital.”
• “Let me know that I won't get COVID if I do get the shots.”

**Table 5:** Results of chi-square tests of independence for individuals who had not received a dose and were deemed either a vaccine acceptor or vaccine refuser based on feedback as to whether they were likely to obtain the COVID-19 vaccine.

Variable	*N* (%)	Respondents who did not receive at least one dose of the COVID-19 vaccine and who either are a vaccine acceptor of vaccine refuser.		Chi-square (df), *P* value
		Acceptor	Refusers	
		*n* (%)	*n* (%)	
Sex	73 (100.0)	27 (100.0)	46 (100.0)	
Female	41 (56.2)	14 (51.9)	27 (58.7)	0.32 (1), *p* = .569
Male	32 (43.8)	13 (48.1)	19 (41.3)	
Age Level	73 (100.0)	27 (100.0)	46 (100.0)	
18–34 years of age	11 (15.0)	7 (25.9)	4 (8.7)	4.22 (2), *p* = .121
35–49 years of age	31 (4.25)	9 (33.3)	22 (47.8)	
50+ year of age	31 (4.25)	11 (40.7)	20 (43.5)	
Race^a^	78 (100)	31 (100.0)	47 (100.0)	
American Indian or Alaskan Native	2 (2.5)	1 (2.2)	1 (3.7)	n/a
Asian	2 (2.6)	2 (7.4)	0 (2.6)	n/a
Black or African American	12 (1.5)	4 (14.8)	8 (17.4)	0.17 (1), *p* = 0.672^e^
Native Hawaiian or Other Pacific Islander	0 (0)	0 (0)	0 (0)	n/a
White	54 (69.2)	23 (85.2)	31 (67.4)	0.743 (1), *p* = .743^e^
Other	8 (10.3)	1 (3.7)	7 (6.2)	n/a
Ethnicity^a^	73 (100.0)	27 (100.0)	26 (100.0)	
Not of Hispanic, Latino, or Spanish origin	52 (71.2)	16 (59.3)	36 (78.3)	2.99 (1), *p* = .083^e^
Hispanic, Latino, or Spanish origin	21 (28.8)	11 (40.7)	10 (21.7)	
Mexican, Mexican American, Chicano	14 (63.7)	6 (22.2)	8 (17.4)	n/a
Puerto Rican	0 (0)	0 (0)	0 (0)	n/a
Cuban	2 (9.1)	1 (3.7)	1 (2.2)	n/a
Other Hispanic, Latino, or Spanish origin	6 (2.3)	4 (14.8)	2 (4.3)	n/a
Education Level	72 (100.0)	27 (100.0)	45 (100.0)	
≤High School Diploma^b^	6 (8.3)	3 (11.1)	3 (6.5)	1.64 (2), *p* = .441
≥HS Diploma < Bachelor's Degree^c^	41 (56.9)	17 (63)	24 (52.2)	
≥Bachelor's Degree^d^	25 (34.7)	7 (25.9)	18 (39.1)	
Annual Household Income	61 (100.0)	22 (100.0)	39 (100.0)	
<$10,000	12 (19.7)	7 (31.8)	5 (12.8)	10.80 (6), *p* = .095
$10,000–$29,999	20 (32.8)	9 (40.9)	11 (28.2)	
$30,000–$39,999	12 (19.7)	1 (4.5)	11 (28.2)	
$40,000–$49,999	3 (4.9)	2 (9.1)	1 (2.5)	
$50,000–$75,999	8 (13.1)	1 (4.5)	7 (17.9)	
$76,000–$99,999	2 (3.3)	1 (4.5)	1 (2.6)	
$100,000 or more	4 (6.6)	1 (4.5)	3 (7.6)	

^a^ Respondents were able to represent their racial and ethnic heritage by selecting more than one racial or ethnic group.

^b^ Includes respondents who self-identified as attaining an education level of “Less than some high school,” “Some high school,” and “High School Diploma, GED, or equivalent”.

^c^ Includes respondents who self-identified as attaining an education level of “Trade school,” “Some college,” and “Associate degree”.

^d^ Includes respondents who self-identified as attaining an education level of “Bachelor Degree” or “Graduate Degree”.

^e^ These samples are independent and based on the appropriate independent sample denominator for the calculation of interest on the appropriate independent sample denominator for the calculation of interest.

n/a: Cannot calculate due to small cell size.

## Limitations

We identify the following limitations to this study:

1. The study population was limited to individuals who purchased ACA health insurance on the open market. Such individuals may exhibit health-seeking behaviors that are different from those who do not have health insurance.
2. We did not validate vaccine uptake by individuals in this survey against Texas immunization registry data.
3. Individuals who respond to a survey from their health insurance company may feel obliged to report positive health-seeking behaviors—regardless of actual behaviors. However, the qualitative feedback from those who did not obtain a COVID-19 vaccine was particularly candid, thus reducing concerns of mis-reported positive health-seeking behaviors.
4. We have applied the Task Force model to a population different from that in which the model was originally designed; as such, findings from our population may differ from that of a healthcare workforce.
5. The authors are responsible for disentangling respondents in the moveable middle to either a vaccine acceptor or vaccine refuser based on subjective interpretation of the qualitative feedback of member responses to the survey.

## Conclusion

Vaccine hesitancy is a complicated construct. Much of the research published on this topic seeks to identify sociodemographic characteristics associated with hesitancy related to one or more specific vaccines or to identify constructs associated with different categories of vaccine uptake. This current study sought to assess the COVID-19 Vaccination Uptake Behavioral Science Task Force for the US Centers for Medicare and Medicaid Services using real-world evidence in Central Texas at six months post vaccine availability. In doing so, we sought to initially quantify individuals into one of three categories: vaccine acceptors, vaccine refusers, and the moveable middle. For those individuals in the moveable middle, we sought to further categorize them into one of the two remaining categories based on the statement, “What, if anything, could be done to change your mind from [*Not sure*] [*Prefer not to answer*] to ‘Yes, I plan to get the COVID-19 vaccine?’” In so doing, we were able to quantify the proportion of individuals who were vaccine acceptors and vaccine refusers at 94.9% (*n* = 854) and 5.1% (*n* = 46), respectively, after the moveable middle was considered. We calculated the moveable middle category at 2.9% (*n* = 26) before reclassification.

What does this mean for public health? It means that there is a decreasing window of opportunity to encourage vaccine acceptance during a public health emergency. This window narrows over time with more and more non-vaccinated people entering the moveable middle. At six months, our data show that most people are committed to being either a vaccine acceptor or a vaccine refuser, with very limited scope for movement between these two groups. Our data also show that for those who are undecided and therefore are in the moveable middle category, when the decision is made to exit, they will most likely do so as a vaccine refuser. Our data show that 84.6% of those in the moveable middle exit as vaccine refusers.

The role of health insurance companies to support a public health emergency response should also not go unnoticed. As evidenced by this study and previous studies from our research team (3, 11, 24), health insurance companies have access to data and a member population that can be accessed when needed to answer pressing questions of public health importance. The COVID-19 pandemic is one such example, and while national and international data can help guide decision-making, it is important to remember the old adage that all disasters are local. Therefore, partnerships within the community can support emerging and ongoing policy related to public health preparedness and response using real-world evidence that is responsive to the needs and expectations of the local community.

## Data Availability

The raw data supporting the conclusions of this article will be made available by the authors, without undue reservation.
